# Lymphoscintigraphy findings in patients with chylothorax: influence of biochemical parameters

**DOI:** 10.1186/s13550-023-01014-0

**Published:** 2023-08-03

**Authors:** Li Zhang, Xiaoyue Zhang, Zhe Wen, Guansheng Tong, Kun Hao, Yongkang Qiu, Lei Kang

**Affiliations:** 1 Department of Nuclear Medicine, Beijing Shijitan Hospital, Capital Medical University, No. 10 Tie Yi Rd, Haidian Dist., Beijing, 100038 China; 2 Department of Lymphatic Surgery, Beijing Shijitan Hospital, Capital Medical University, Beijing, 100038 China; 3 Department of Nuclear Medicine, Peking University First Hospital, No. 8 Xishiku Str., Xicheng Dist., Beijing, 100034 China

**Keywords:** Lymphoscintigraphy, Chylothorax, Triglyceride, Cholesterol, Lactate dehydrogenase

## Abstract

**Background:**

Chylothorax is a condition that can be challenging to diagnose due to its nonspecific clinical presentation. Several biochemical parameters of chylous pleural effusion have been identified as important indicators for the diagnosis of chylothorax. Lymphoscintigraphy is utilized to assess chylothorax and determine the location of chyle leakage. The present study aimed to evaluate the correlation between the biochemical parameters of chylous pleural effusion and ^99m^Tc-dextran (^99m^Tc-DX) lymphoscintigraphy in diagnosing chylothorax.

**Material and methods:**

A total of 120 patients were enrolled in the study, 83 of the patients with unilateral chylothorax, and 37 with bilateral chylothorax. All patients underwent both ^99m^Tc-DX lymphoscintigraphy and pleural effusion laboratory analysis. The ^99m^Tc-DX lymphoscintigraphy images were categorized as positive or negative groups based on the presence or absence of abnormal radioactive tracer accumulation in the thorax, respectively. The biochemical parameters of the two groups were subsequently compared.

**Results:**

Among these patients, 101 (84.17%) had exudative effusions, while 19 (15.83%) had transudative effusions, as determined by the levels of pleural effusion protein, lactate dehydrogenase and cholesterol. Abnormal tracer accumulation in thorax was observed in 82 patients (68.33%). Our findings indicated that lymphoscintigraphy results were not associated with exudative and transudative chylothorax (*P* = 0.597). The lymphoscintigraphy positive group displayed significantly higher levels of pleural effusion triglyceride and pleural effusion triglyceride/serum triglyceride ratio in all biochemical parameters, compared to the negative group (*P* = 0.000 and *P* = 0.005). We identified cutoff values of 2.870 mmol/L for pleural effusion triglycerides and 4.625 for pleural effusion triglyceride/serum triglyceride ratio, respectively, which can facilitate differentiating the positive and negative cases on lymphoscintigraphy.

**Conclusion:**

Lymphoscintigraphy technique is a dependable diagnostic tool for the qualitative assessment of chylous pleural effusion. Higher pleural effusion triglyceride level and pleural effusion triglyceride/serum triglyceride ratio indicate a positive result in patients with chylothorax on lymphoscintigraphy, with the cutoff values of 2.870 mmol/L and 4.625 aiding in the diagnosis.

## Introduction

Chylothorax is an infrequent medical condition characterized by the leakage of chyle from the lymphatic system into the pleural space [1]. Chyle, a milky fluid composed of triglycerides, protein, lymphocytes, and immunoglobulins, is produced in the lacteal system of the intestine and transported to the bloodstream via the thoracic duct [2–4]. The diagnosis of chylothorax is typically made upon the identification of milky pleural effusion. [5–9]. Unfortunately, fewer than 50% of the chylothorax cases present with this classic appearance of pleural effusion [10]. The untimely identification of chylothorax may lead to persistent depletion of chyle fluid, ultimately resulting in malnourishment, compromised immunity, metabolic disorders, and even fatality [11, 12].

Accurate diagnosis is imperative for the efficacious management of chylothorax progression [13]. Diagnostic process entails an examination of the pleural fluid, whereby a chylous effusion typically exhibits elevated triglyceride levels exceeding 110 mg/dl, reduced cholesterol levels below 200 mg/dl, and the presence of chylomicrons [10, 14, 15]. The identification of chylomicrons in the pleural fluid through lipoprotein electrophoresis serves as the definitive diagnostic criterion for chylothorax. Nonetheless, it should be noted that certain medical institutions may lack the capability to perform the lipoprotein electrophoresis test. Furthermore, most (80%) chylous pleural effusions are likely to be classified as exudative according to the Light’s criteria [10, 14]. And the pleural fluid to serum triglyceride ratio should be greater than 1 and the pleural fluid to serum cholesterol ratio less than 1 [14].

Lymphoscintigraphy has emerged as a prevalent and efficacious approach for qualitative diagnosis of chylothorax and identifying the location of the ruptured sites of the lymph vessel [5, 16–18]. This imaging technique is convenient, minimally invasive, and devoid of any known adverse effects [19, 20]. As an imaging agent for lymphoscintigraphy, ^99m^Tc-dextran (^99m^Tc-DX) is a polysaccharide nonprotein macromolecule with varying molecular weight. It metabolized by the liver and excreted by the kidneys. Its rapid uptake from the injection site is a notable advantage. In the context of chylothorax, the tracer can traverse damaged lymphatic vessels and enter the pleural cavity, facilitating effusion visualization. Despite several case reports indicating the diagnostic potential of lymphoscintigraphy for chylothorax [21, 22], the impact of laboratory examination parameters(e.g., triglycerides, cholesterol, and proteins, etc.) on its results remains unclear. In this study, we conducted an investigation into the diagnostic efficacy of ^99m^Tc-DX lymphoscintigraphy in identifying of chylothorax patients, as well as exploring the potential correlation between ^99m^Tc-DX lymphoscintigraphy and the biochemical parameters of chylous pleural effusion.

## Material and methods

### Population

**Fig. 1:**
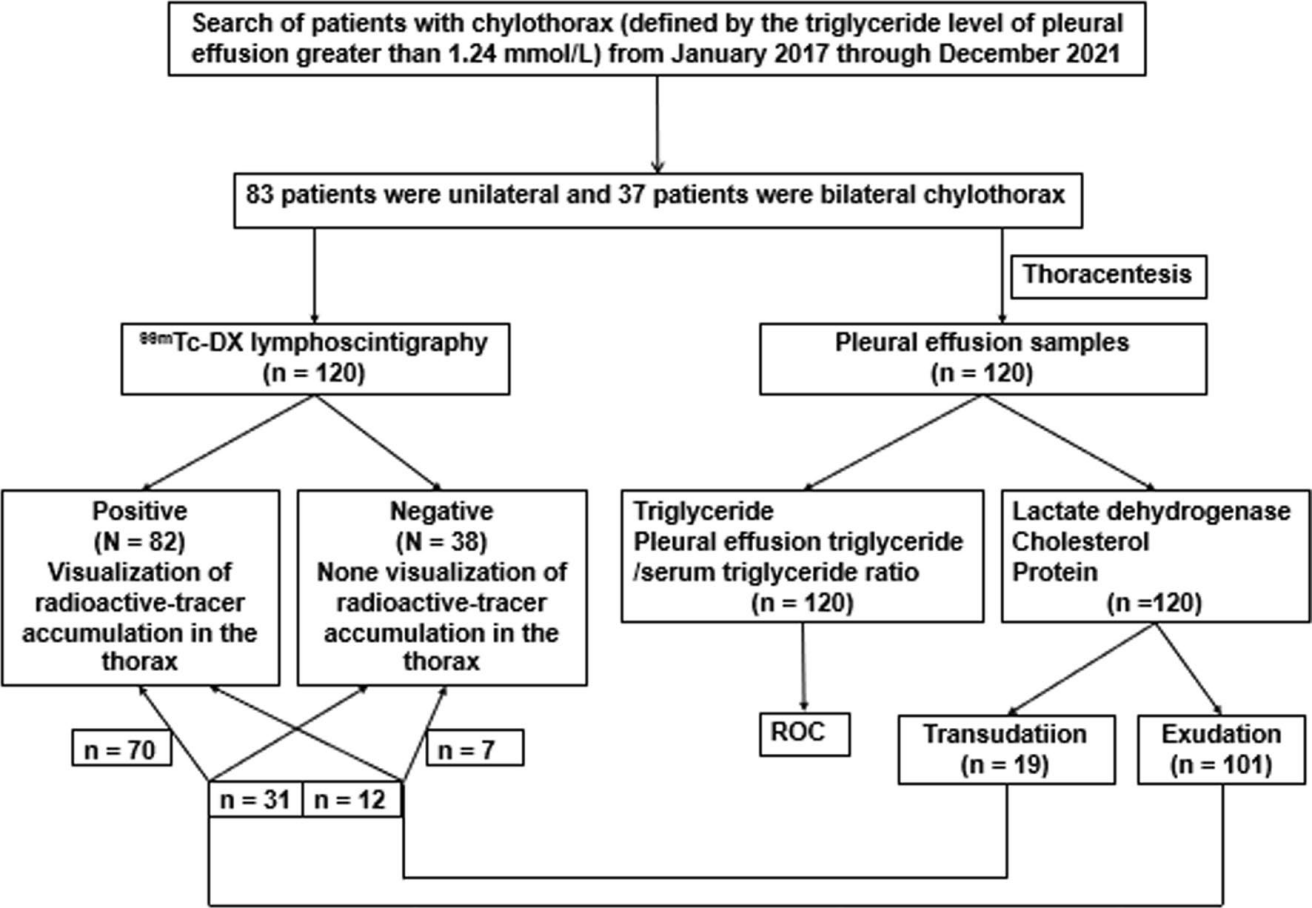
Flowchart of patient selection for this retrospective case‒controlled study

All patients with chylothorax who underwent ^99m^Tc-DX lymphoscintigraphy and pleural effusion laboratory analysis from January 2017 to December 2021 in the Department of Nuclear Medicine of Beijing Shijitan Hospital Affiliated with Capital Medical University were retrospectively analyzed. The inclusion criteria for chylothorax were the triglyceride level of pleural effusion greater than 1.24 mmol/L. All patients with pleural effusion underwent thoracocentesis or drainage to extract pleural effusion samples, which were then subjected to laboratory biochemical tests. The median time interval between lymphoscintigraphy and pleural effusion laboratory analysis was 6 days (range, 0–30 days). In the case of chylothorax patients, the blood biochemical parameters and pleural effusion laboratory analysis were conducted simultaneously. The medical records were reviewed by the electronic medical record system, including clinical data, laboratory data, and the results of ^99m^Tc-DX lymphoscintigraphy as well as operative notes (Fig. 1).

### Lymphoscintigraphy imaging

Images were acquired with a dual-head gamma-camera with a low-energy, high-resolution collimator in whole-body scanning. The parameters of the scan included an energy peak of 140 keV, window width of 20%, acquisition matrix of 256 × 1024 pixel, and zoom of 1 at a scan speed of 17 cm/min. Single-photon emission computed tomography/computed tomography (SPECT/CT) (Symbia T16, Siemens Medical Systems, Germany) fusion images of the main affected area were obtained 3 h after the injection of radiotracer. SPECT imaging was performed using a 128 × 128 matrix with a spacing of 3.0 mm, 62 projections over 360°, and 45 s per projection. CT scan of the same anatomical region was obtained with 130 kV, 190mAs, and pitch 0.65. Perform iterative reconstruction of the SPECT data using a Flash 3D algorithm; 8 iterations and 4 subsets. ^99m^Tc-DX (Beijing Atomic Technology Co., Ltd.) was injected subcutaneously at two points on each foot, one between the first and second toes and another between the fourth and fifth toes. The dose of each injection point was 55.5–92.5 MBq, and the injection volume was 0.1–0.15 mL. Whole-body images from head to foot were acquired at 10 min, 1 h, 3 h, and 6 h postinjection.

### Image analysis

The lymphoscintigraphy images were analyzed by two independent, experienced nuclear medicine physicians who reached a consensus on each scan. Analysis of the imaging characteristics of the scans first determined whether the lymphatic drainage was normal. Normal lymphatic drainage was defined as normal uptake of linear lower limbs lymphatic ducts and ilioinguinal lymph nodes without backflow in the subcutaneous tissues of the lower limbs and trunk. Second, and more importantly, the analysis focused on whether there was persistent visualization of radioactive accumulation in the thorax corresponding to the known chylothorax suggesting a chylous pleural effusion and regarded as a positive result on lymphoscintigraphy. Abnormal radioactive accumulation was defined as that the radioactivity on the affected side is higher than that on the healthy side or gradually increases with time delay. Otherwise, it was considered as a negative result. If there was a focal accumulation of radiotracer in the mediastinum or the affected pleural cavity, it suggested a suspected site of chyle leakage. Whether there was abnormal radioactive-tracer distribution in other areas, such as the abdominal cavity, was also analyzed.

### Pleural effusion evaluation

Chylous pleural effusions were classified as exudation or transudation according to following criteria [10]. A chylous pleural effusion was exudative if one of the following conditions was met: (1) pleural effusion protein level greater than 29 g/L, (2) pleural effusion lactate dehydrogenase (LDH) level greater than two-thirds of the upper limit of the normal serum value (the normal serum LDH level referred to 120 U/L to 250 U/L), and (3) pleural effusion cholesterol level greater than 1.166 mmol/L. Chylous effusion was classified as transudation if none of the above conditions were met or if the following two conditions were met: (1) pleural effusion LDH level two-thirds or less than the upper limit of the normal serum value; (2) pleural effusion cholesterol level of 45 mg/dL or less.

### Statistical analysis

Statistical analysis was performed using SPSS Statistics (Version 24.0, IBM). Chylous effusion biochemical parameters between the positive and negative groups on lymphoscintigraphy were compared using an independent *t* test or nonparametric tests. Comparison of lymphoscintigraphy results between the exudative chylothorax and transudative chylothorax was performed using the Pearson Chi-square test. The results were considered to be statistically significant when the *P* value was less than 0.05. If there was a significant difference in chylous effusion laboratory analysis between the positive and negative groups on lymphoscintigraphy, additional receiver operating characteristic (ROC) curve analysis was performed to determine the area under the curve (AUC) value. The optimal cutoff value was calculated, as well as the sensitivity, specificity, and accuracy.

## Results

### Population study

This study retrospectively collected 120 patients, including 57 males (47.5%) and 63 females (52.5%), with a median age of 41.0 years (range, 1–77 years). Chylothorax was unilateral in 83 patients (69.17%). Of these 83 patients, the chylothorax involved the right hemithorax in 52 patients (6 cases with ascites) and the left hemithorax in 31 patients (2 cases with ascites). Chylothorax was bilateral in the remaining 37 patients (30.83%) (6 cases with ascites). Of the 37 patients with bilateral pleural effusion, laboratory examinations were performed on one side of the pleural effusion samples. Patient’s clinical symptoms were documented in 101 patients (84.17%). Dyspnea was the most common symptom, presenting in 72 patients. Cough was presented in 8 patients, abdominal distension in 14 patients, and limb swelling in 7 patients. The reason of chylothorax was idiopathic in 62 patients (51.67%), lymphatic disorder in 34 patients (28.33%), surgery and trauma in 10 patients (8.33%), malignancies in 4 patients (3.33%), and others in 10 patients (8.33%).

### Lymphoscintigraphy performance

**Fig. 2:**
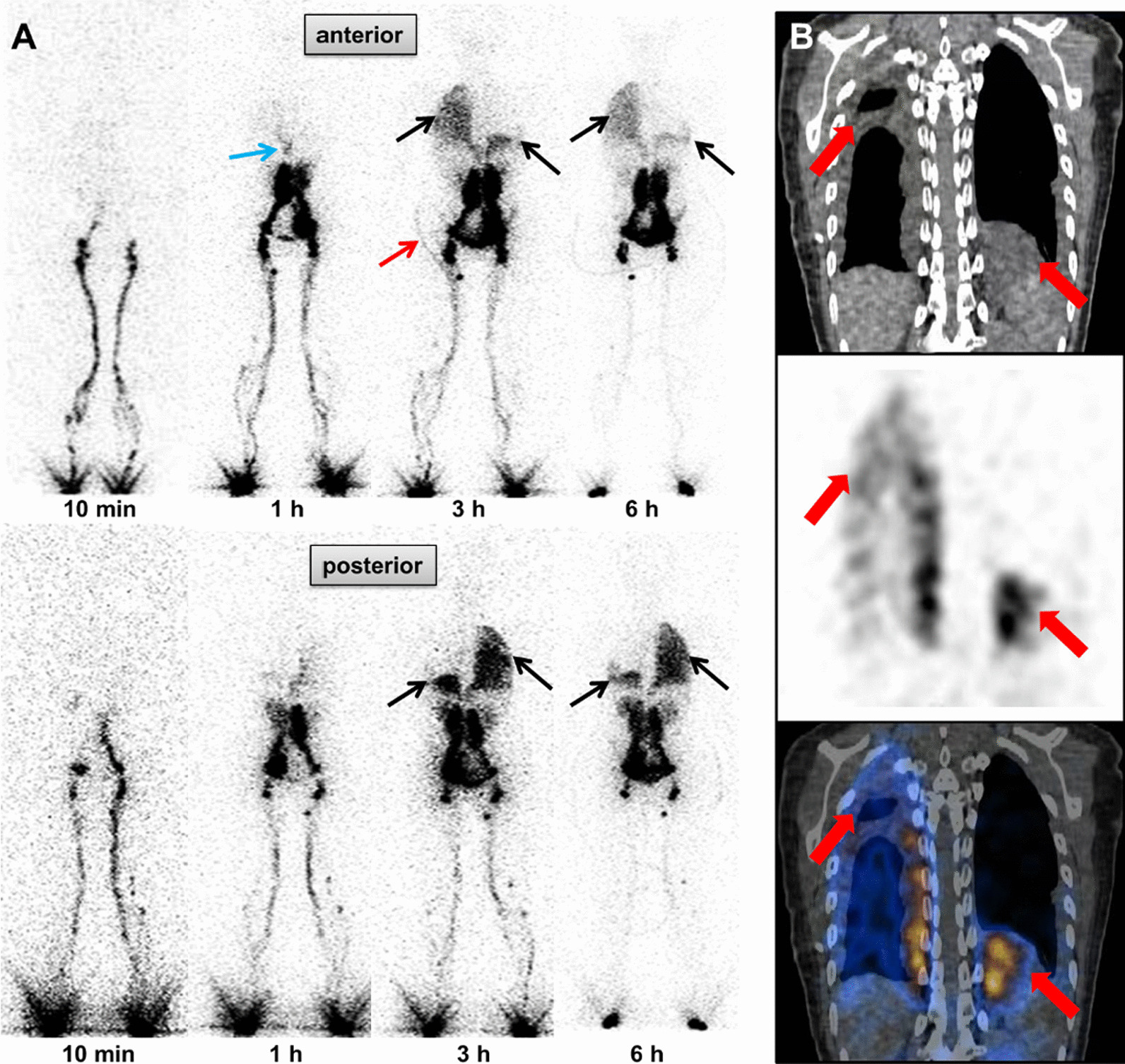
A 29-year-old female with idiopathic chylothorax (bilateral). A continuous drainage tube was placed in the right pleural cavity, and laboratory analysis confirmed the right pleural effusion as a transudation. **A** The anterior (upper row) and posterior (lower row) planar images of the lymphoscintigraphy were acquired at 10 min–6 h. A localization site of lymphatic leakage was seen in the thoracic duct region (diaphragmatic level) at 1 h anterior (blue arrow). Radioactive tracer could be seen in the right thoracic drainage tube at 3 h anterior (red arrow). Visualization of radioactivity was observed in the left lower thorax and the right thorax at 3–6 h, suggesting a chylothorax on both sides (black arrows). **B** The thorax region SPECT/CT images (upper row CT image; middle row SPECT image; lower row hybrid SPECT/CT image) acquired at 3 h. The focal accumulation of radioactive tracer in the thoracic duct region had disappeared. Abnormal activity was seen in well-defined pleural effusion in the left lower thorax and the right thorax (thick red arrows)

**Fig. 3:**
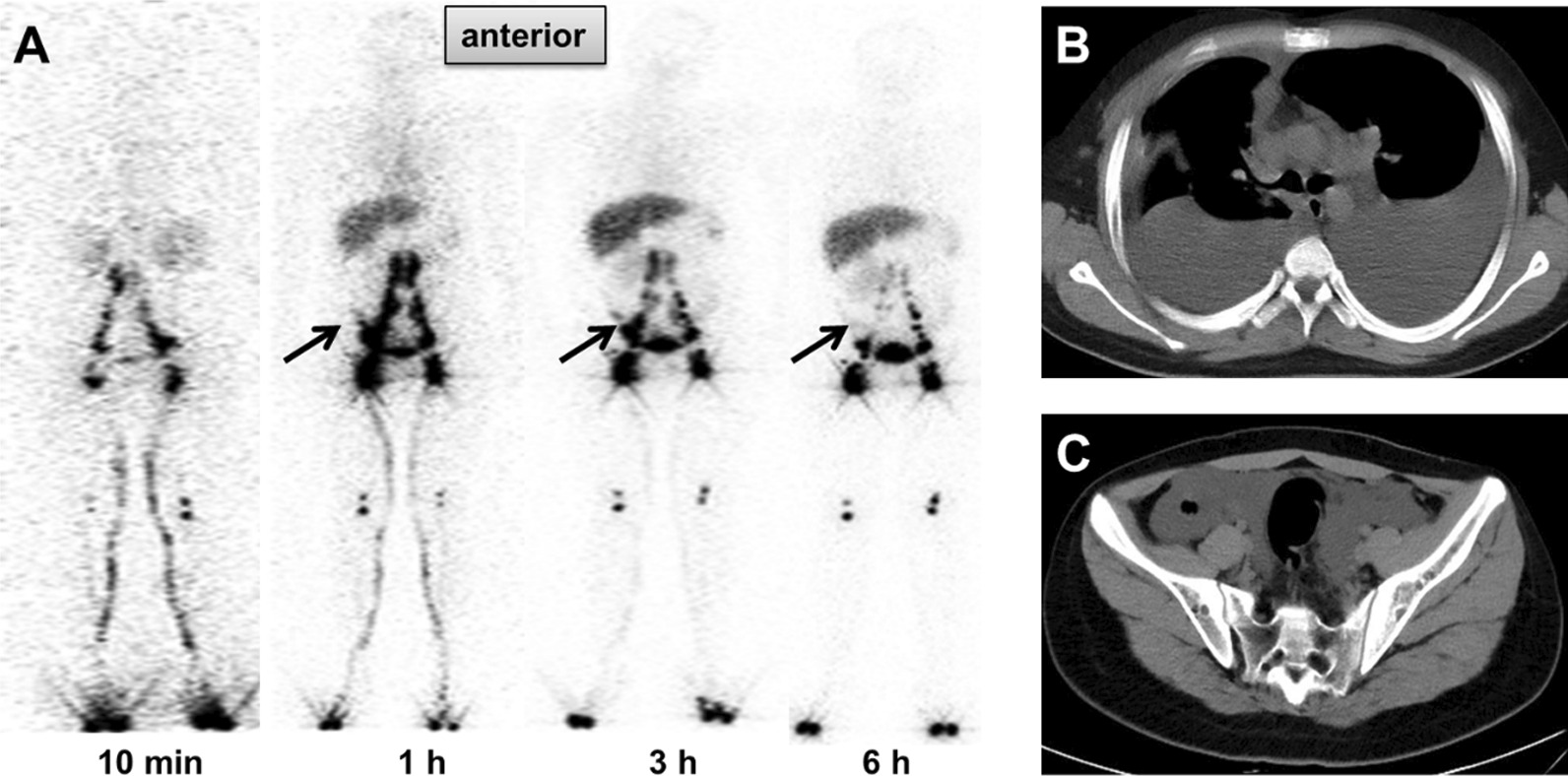
A 14-year-old male with bilateral pleural effusion and ascites. The right-sided pleural effusion confirmed exudative chylothorax. **A** The anterior lymphoscintigraphy images acquired at 10 min–6 h showed no elevated radioactivity in the bilateral thoracic cavities and arc-shaped abnormally elevated radioactivity in the pelvic cavity (black arrows). **B**, **C** The CT aerial views of the chest and pelvis showed massive liquid in the bilateral thoracic cavities and abdominal cavity

**Figure. 4:**
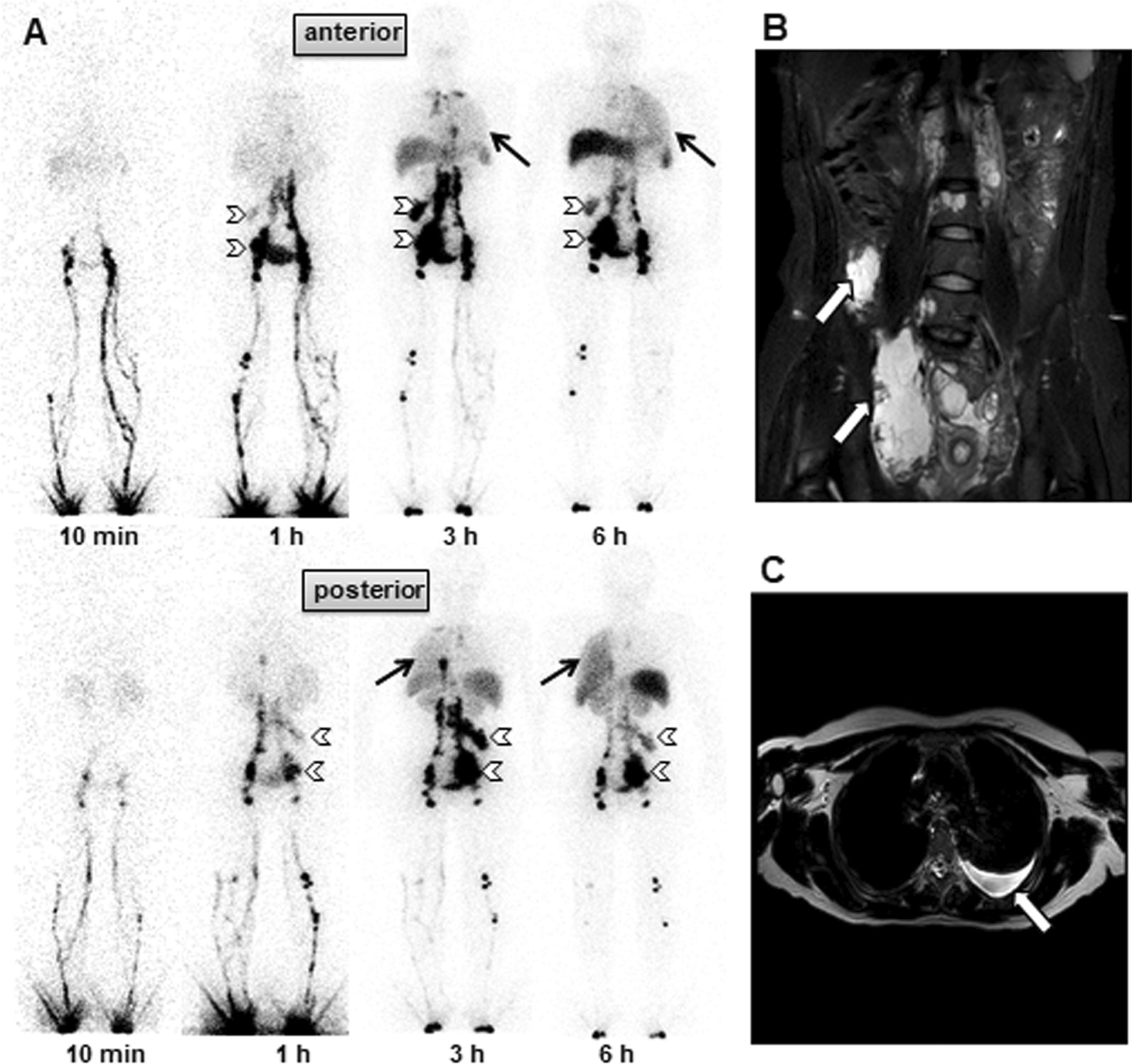
A 29-year-old female with lymphangioma and left exudative chylothorax. **A** The anterior (upper row) and posterior (lower row) planar images of the lymphoscintigraphy were acquired at 10 min–6 h. The visualization of radioactivity was observed in the left hemithorax at 3–6 h anterior and posterior, suggesting a chylothorax (black arrows). Two focal accumulations in the right lower abdomen were observed (empty arrowheads) corresponding to lymphangioma on MRI scan (empty arrows) **B**. **C** An MRI scan revealed left-sided pleural effusion (empty arrow)

Of these 83 patients with unilateral chylothorax, 54 patients showed persistent radioactive-tracer accumulation on the affected side on lymphoscintigraphy imagings, while the remaining 29 patients did not. In the cases of the 37 patients with bilateral chylothorax, 28 displayed persistent radioactive-tracer accumulation on both sides, while 9 exhibited no visually abnormal activity. Two patients were identified with suspected sites of chyle leakages (Fig. 2). Additionally, 7 patients with known limb swelling presented with abnormal activity in the lower extremity. Two patients exhibited imaging findings of limb lymphedema despite the absence of subjective symptoms of limb swelling. Among the 14 patients presenting with both chylothorax and ascites, 9 showed abnormal radioactive tracer accumulation in both the thoracic and abdominal cavities, while 5 showed elevated radioactivity in the abdominal cavity but not in the thoracic cavity (Fig. 3). Of the 14 patients with lymphangioma, lymphangioma was visualized in only one patient (Fig. 4).

### Correlation between laboratory analysis and lymphoscintigraphy

**Fig. 5:**
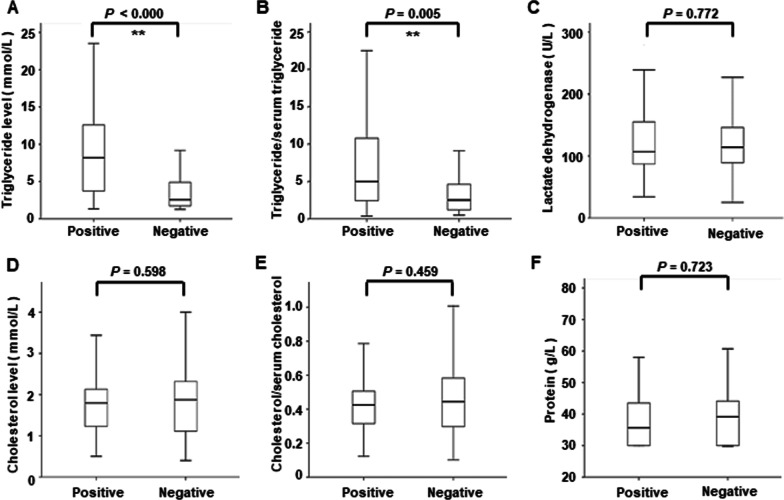
Laboratory examination characteristics between positive and negative groups shown by lymphoscintigraphy. **A** Pleural effusion triglyceride; **B** Pleural effusion triglyceride/serum triglyceride ratio; **C** Pleural effusion lactate dehydrogenase; **D** Pleural effusion cholesterol; **E** Pleural effusion cholesterol/serum cholesterol ratio; **F** Pleural effusion protein

Medical records for 120 chylous pleural effusion samples revealed that 66 samples (55.0%) were described as milky or bloody chyle, and 54 samples (45.0%) were not chyle, which could be bloody, orange, yellow or straw-colored. For all the biochemical parameters of chylous pleural effusion, statistically significant differences were observed in the median of triglyceride level and pleural effusion triglyceride/serum triglyceride ratio between the positive and negative groups on lymphoscintigraphy (*P* = 0.0001; *P* = 0.005) (Fig. 5). In accordance with the aforementioned standards, 101 samples (84.17%) were classified as exudative, while 19 samples (15.83%) were categorized as transudative. The etiologies of the 19 transudative chylothoraces included liver cirrhosis (4 patients), surgery (2 patients), lymphatic disorder (8 patients), and idiopathic cause (5 patients). However, there was no correlation between chylous pleural effusion types and lymphoscintigraphy results (*P* = 0.597) (Table 1).

**Table 1:** Correlation between chylous pleural effusion types and lymphoscintigraphy

	Lymphoscintigraphy of Chylothorax		Total	*χ*^2^	*P*
	Positive	Negative			
Exudation	70	31	101	0.279	0.597
Transudation	12	7	19		
Total	82	38	120		

### Performance of laboratory analysis for predicting lymphoscintigraphy

**Fig. 6:**
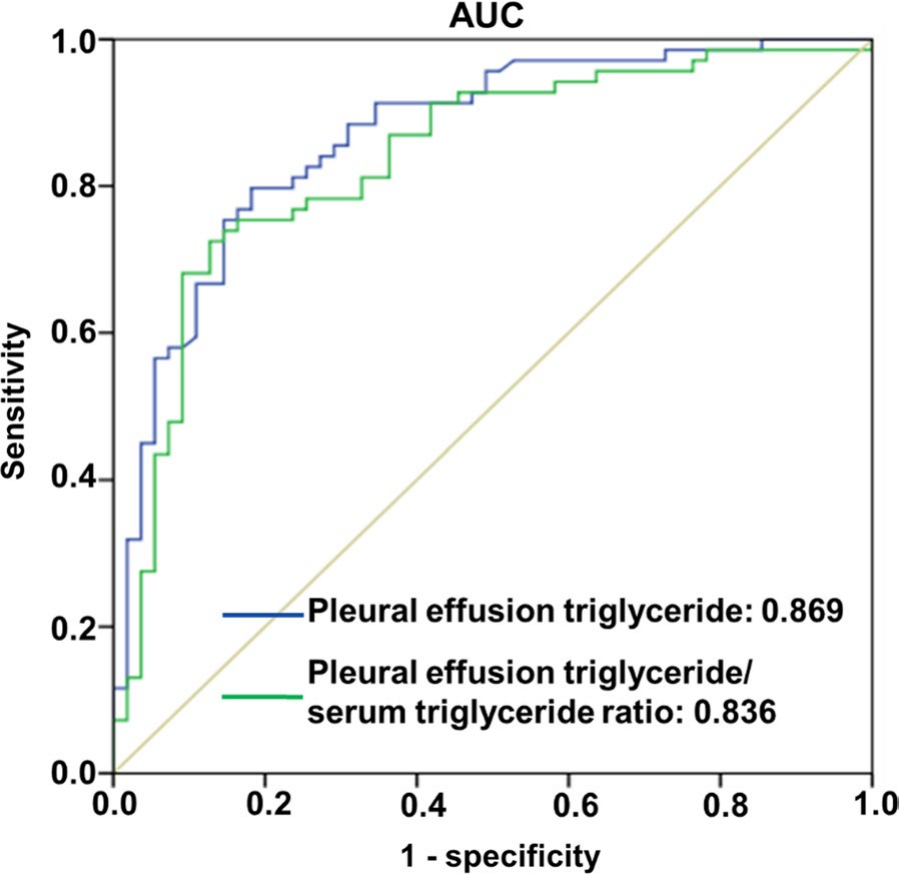
ROC curves. ROC curves analyses suggested both pleural effusion triglyceride and pleural effusion triglyceride/serum triglyceride ratio could predict lymphoscintigraphy

When the chylous pleural effusion triglyceride cutoff value from the ROC curve for lymphoscintigraphy was set to 2.870 mmol/L (ROC AUC, 0.869; 95% CI, 0.805–0.932; *P* = 0.0001), the sensitivity, specificity, and accuracy were 88.4%, 63.2%, and 75.8%, respectively. When the pleural effusion triglyceride/serum triglyceride ratio cutoff value from the ROC curve for lymphoscintigraphy was set to 4.625 (ROC AUC, 0.836; 95% CI, 0.763–0.910; *P* = 0.005), the sensitivity, specificity, and accuracy were 53.5%, 76.3%, 64.9%, respectively (Fig. 6).

## Discussion

All pleural effusions with a triglyceride level exceeding 1.24 mmol/L have been demonstrated to be chylothorax [14, 23–25]. The composition of chyle is contingent upon dietary factors, and an increase in lymph flow through the thoracic duct is observed during the absorption phase of digestion, making the chyle liquid show the typical milky appearance [26–29]. Conversely, a decrease in lymph flow during fasting may result in chyle effusion having a clear appearance. Milky effusions have been found to have significantly higher levels of triglycerides compared with non-milky effusions, although other fluid characteristics, such as protein, LDH, and cholesterol levels, were similar [10]. Therefore, the examination of pleural effusion for triglycerides plays an important role in diagnosing chylothorax.

Lymphoscintigraphy is considered as the gold standard for the diagnosis of lymphedema and can be used to evaluate chylothorax in patients even with dyspnea [30]. In our study, we found that 68.33% of patients with chylothorax had an abnormal accumulation of radioactive tracer in the thorax during lymphoscintigraphy. Furthermore, our analysis of the biochemical parameters of chylous pleural effusion revealed that only triglyceride and pleural effusion triglyceride/serum triglyceride ratio showed a significant difference in the lymphoscintigraphy positive group and negative group. The cutoff values of pleural effusion triglyceride and pleural effusion triglyceride/serum triglyceride ratio are 2.870 mmol/L and 4.625, respectively, which is helpful in indicating lymphoscintigraphy results in patients with chylothorax. Interestingly, the cutoff value of triglyceride for suggesting lymphoscintigraphy results was greater than the standard value used for laboratory diagnosing chylous effusion. This discrepancy may be explained by the fact that some patients switched from a normal diet to a high-fat diet after negative results were found on lymphoscintigraphy, which may have stimulated chyle activity in the effusion [31]. It should be noted that when chylothorax coexists with chylous ascites and too many radioactive tracer leaks into the peritoneal cavity, it may lead to insufficient tracer to visualize the chylothorax. However, ^99m^Tc-DX lymphoscintigraphy was not ideal for identifying the location of lymphatic leakage, with only two patients in the study showing suspicious chyle leakage points.

Chylothorax is nearly always exudative. The incidence of the exudative condition in our chylothorax patient accounts for 84.17%, which is consistent with previous reports [10, 32]. Transudative chylothorax is extremely rare and mostly is associated with hepatic cirrhosis, nephrotic syndrome, amyloidosis, and obstruction of the superior vena cava [11, 33–35]. In our study, typical causes of such transudative chylothorax were found in 19 patients, including hepatic cirrhosis (4 patients), surgery (2 patients), lymphatic disorder (8 patients), and idiopathic (5 patients). These observations were not completely similar to what was reported previously [11]. Exudative and transudative chylothorax were only related to the levels of proteins, LDH, and cholesterol in the pleural effusion [10], which were not significantly different in the lymphoscintigraphy positive group and negative group. There was no correlation between chylous pleural effusion types and results on lymphoscintigraphy. Therefore, it can be concluded that the lymphoscintigraphy results of chylothorax are not affected by the types of chylothorax.

Our results showed that lymphoscintigraphy not only confirmed chylothorax in most patients but also found extra thorax lymphatic system lesions. Such example is lymphangiomas, which are rare, benign lymphatic pathological change that arise due to the failure of the individual lymphatic tissues to connect with the general lymphatic system, and may or may not communicate with the rest of the system [36–38]. In our study, ^99m^Tc-DX lymphoscintigraphy did not find as many lesions as reported in the literature [39]. Only one patient with lymphangioma exhibited radioactive accumulation at the focus during lymphoscintigraphy. In addition, lymphoscintigraphy detected all lymphedema in our study.

There are several limitations to our study. Only the chylothorax patients with pleural effusion triglyceride levels greater than 1.24 mmol/L were included in the study, despite evidence indicating that 14% of pleural effusions may be related to chylothorax even when triglyceride levels are below this threshold [11]. Furthermore, while lipoprotein electrophoresis considered the gold standard for diagnosis of chylothorax should be conducted to select patients, this test was not available at our institute [24]. Consequently, patients with pleural effusion triglyceride levels below 1.24 mmol/L were excluded from the study, which may have led to an inevitable selection bias. In addition, we did not specify the diet of patients before imaging. Those chylothorax patients who are fasting might affect the results of lymphoscintigraphy.

## Conclusion

In conclusion, ^99m^Tc-DX lymphoscintigraphy is convenient to be performed in patients of any age, including neonates, and it can be repeated in a short interval. It is a reliable diagnostic tool for qualitative evaluation of chylous pleural effusion. In patients who decline thoracocentesis, lymphoscintigraphy can also serve as a qualitative diagnostic tool for chylothorax. Higher pleural effusion triglyceride level and pleural effusion triglyceride/serum triglyceride ratio suggest a positive result in patients with chylothorax on lymphoscintigraphy, with cutoff values of 2.870 mmol/L and 4.625 improving diagnosis potentially. According to the findings of this study, it is advisable to defer lymphoscintigraphy for individuals suspected of chylothorax whose pleural effusion triglyceride levels are less than 2.870 mmol/L to prevent erroneous negative outcomes. Furthermore, lymphoscintigraphy can also effectively assess rare lymphatic disorders such as lymphedema and lymphangioma in chylothorax patients.

## Data Availability

The datasets analyzed during the current study are not publicly available due relevant data protection laws.

## Abbreviations

^99m^Tc-DX: Technetium (^99m^Tc) dextran
ROC: Receiver operating characteristic
LDH: Lactate dehydrogenase
AUC: Area under the curve
MRI: Magnetic resonance imaging
SPECT: Single-photon emission computed tomography
